# Malaria in gold-mining areas in Colombia

**DOI:** 10.1590/0074-02760150382

**Published:** 2016-01

**Authors:** Angélica Castellanos, Pablo Chaparro-Narváez, Cristhian David Morales-Plaza, Alberto Alzate, Julio Padilla, Myriam Arévalo, Sócrates Herrera

**Affiliations:** 1 Malaria Vaccine and Drug Development Centre, Cali, Colombia; 2 National Institute of Health of Colombia, Bogotá, Colombia; 3 Caucaseco Scientific Research Centre, Cali, Colombia; 4 Ministry of Health and Social Protection, Bogotá, Colombia; 5 Universidad del Valle, Faculty of Health, Cali, Colombia

**Keywords:** mining, malaria, Plasmodium vivax, Plasmodium falciparum, gold

## Abstract

Gold-mining may play an important role in the maintenance of malaria worldwide. Gold-mining, mostly illegal, has significantly expanded in Colombia during the last decade in areas with limited health care and disease prevention. We report a descriptive study that was carried out to determine the malaria prevalence in gold-mining areas of Colombia, using data from the public health surveillance system (National Health Institute) during the period 2010-2013. Gold-mining was more prevalent in the departments of Antioquia, Córdoba, Bolívar, Chocó, Nariño, Cauca, and Valle, which contributed 89.3% (270,753 cases) of the national malaria incidence from 2010-2013 and 31.6% of malaria cases were from mining areas. Mining regions, such as El Bagre, Zaragoza, and Segovia, in Antioquia, Puerto Libertador and Montelíbano, in Córdoba, and Buenaventura, in Valle del Cauca, were the most endemic areas. The annual parasite index (API) correlated with gold production (R^2^ 0.82, p < 0.0001); for every 100 kg of gold produced, the API increased by 0.54 cases per 1,000 inhabitants. Lack of malaria control activities, together with high migration and proliferation of mosquito breeding sites, contribute to malaria in gold-mining regions. Specific control activities must be introduced to control this significant source of malaria in Colombia.

Mining has historically played an important role in the expansion and creation of many productive human settlements and to the national economy of mineral rich countries, but simultaneously it has led to an increase in malaria transmission in mining areas (Knoblauch et al. 2014). African and Asian countries, such as Ghana, South Africa, and Papua New Guinea (PNG), report an important percentage of malaria cases originating in gold-mining areas. Likewise in the American continent, Brazil, Colombia, Venezuela, Suriname, and Peru are countries with significant gold extraction associated with high malaria prevalence (Asante et al. 2011, da Silva-Nunes et al. 2012,Ferreira et al. 2012, Mitjà et al. 2013, Parker et al. 2013), which presents mainly as asymptomatic cases and in age groups involved in mining (de Andrade et al. 1995, da Silva-Nunes et al. 2012).

In countries like Ghana, the overall malaria prevalence was 22.8% in 2006/2007, with ~98% in mining areas that were predominantly *Plasmodium falciparum*infections (Asante et al. 2011), meanwhile in PNG (Lihir Island) *Plasmodium vivax* was more prevalent (57%) in 2006-2011, with a small number of *Plasmodium malariae* cases (< 3%) (Mitjà et al. 2013).

Most malaria cases in Brazil come from rural areas related to gold-mining in the Amazon Region, where 52% of the cases are caused by *P. vivax*, 30% by*P. falciparum*, and the rest are mix infections and *P. malariae* (de Oliveira et al. 2013); the state of Mato Grosso gold-mining contributed a significant number of cases. In 1992, the annual parasite index (API) was 96.1 per 1,000 inhabitants, but between 1993-2002 it decreased to 2.7 cases per 1,000 inhabitants (Ferreira et al. 2012) due to aggressive active case detection (ACD) implemented by the Brazilian government.

In Peru and Suriname the contribution of mining to malaria prevalence appears to be much lower (3-7%) (Villegas et al. 2010, da Silva-Nunes et al. 2012, Parker et al. 2013). In the Amazon regions of Peru, Guyana, and Suriname (Guyana Shield) there has been an increase in malaria transmission mainly in informal mining camps due to the lack of opportune diagnosis, availability, and poor quality of antimalarials (Parker et al. 2013,Pribluda et al. 2014).

In Suriname, 66% of the miner population are Brazilian immigrants (Adhin et al. 2014) and malaria transmission decreased from 14,403 in 2003 to 1,371 in 2009 due to the introduction of artemisinin combination treatment. Gold miners are now the only remaining population that is vulnerable to malaria (Hiwat et al. 2012, Breeveld et al. 2012).

In Colombia mining has for centuries offered the only means of subsistence to some populations, particularly in areas with little presence of the State (Defensoría del Pueblo de Colombia 2010, Semana 2015). However, the Colombian mining industry has grown quickly during the past decade, mostly due to government policies which have favoured foreign investment in mining (PwC 2012), but also because there has been a rapid proliferation of illegal mines (the majority of gold mines are believed to be illegal) (Güiza & Aristizabal 2013). An important example of illegal mining is that of Segovia and Remedios (department of Antioquia), one of the most productive districts, where 348 units of gold-mining have been reported, of which only 14 were legal (MINMINAS 2011, INDEPAZ 2013). Although there is no evidence of an association between malaria and the legal mining activities, it could be presumed that legal mining is more environment-friendly with less artificially man-made mosquito breeding sites. Colombia has a great diversity of *Anopheles* mosquitoes, several of which are either confirmed or suspected malaria vectors. This, together with substantial migration that is frequently induced by mining activities, favours the circulation of malaria infected individuals through mining districts. Moreover, anopheles mosquitoes breed in a great variety of different conditions and adapt to local environmental characteristics such as altitude, climate, weekly rainfall intensity (which influences larval abundance), and land use which may create temporary or permanent man-made habitats in open sky gold-mining. These conditions can significantly impact the phenology and population dynamics of mosquito larvae populations and indirectly affect the dynamics of mosquito-borne diseases (Imbahale et al. 2011). Additionally, the lack of health promotion and prevention measures, the ignorance of such measures by miners, the proximity of their accommodation, known as gold-mining huts, and subsequent migration of workers to other areas, are all factors contributing to the spread of disease, particularly malaria. In malaria endemic communities, a significant percentage (5-15%) of the population usually harbour malaria infections without showing clinical symptoms (Vallejo et al. 2015). These individuals represent a pool of parasites for malaria transmission and thus perpetuate its spread (Moreno et al. 2007, 20, 2009, da Silva-Nunes et al. 2012). Importantly, gold production has been reported to closely correlate with malaria burden. In a previous study it was estimated that for every 100 kg of gold production the API in mining areas would increase by 0.37/1,000 inhabitants, and the annual incidence rates between 160-260 cases/1,000 inhabitants (Duarte & Fontes 2002). The aim of this study was to assess the current malaria situation in Colombia in regions with gold-mining activities and the correlation between gold production and malaria incidence.

## MATERIALS AND METHODS

*Study design* - A descriptive and retrospective study of malaria prevalence in mining areas was carried out based on official information from mining districts, that included 47 endemic municipalities of Antioquia, Bolívar, Córdoba, Chocó, Valle del Cauca, and Nariño departments from 2010-2013 (Table I).

**TABLE I:** Gold-mining district (GMD)

Mining district (department)	Municipalities
Frontino (Antioquia)	Carmen de Atrato^*a*^, Buriticá, Frontino, Abriaquí, Urrao, Dabeiba, Anzá, Mutatá
Northeat Antioqueño (Antioquia)	Amalfi, El Bagre, Segovia, Zaragoza, Remedios, San Roque
Santa Rosa (Bolívar)	Santa Rosa del Sur, Simití, San Pablo
San Martín de Loba (Bolívar)	San Jacinto de Achí, Tiquisio, Morales, San Martín de Loba
Montelíbano (Córdoba)	Buenavista, Planeta Rica, Pueblo Nuevo, La Apartada, Puerto Libertador, Montelíbano
Minero Istmina (Chocó)	Itsmina, Condoto, Tadó, Sipí, Bagadó
Costa Pacífica (Cauca y Valle)	López de Micay, Guapi, Timbiquí, Buenaventura
Costa Pacífica Sur (Nariño)	Barbacoas, Santa Barbara (Iscuandé), Magui Payan
La Llanada (Nariño)	La Llanada, Santa Cruz, Los Andes (Sotomayor), Cumbitara, Samaniego, Mallama (Piedrancha)
Mercaderes (Cauca and El Tambo Nariño)	Bolívar, Tambo

*a*: municipality belonging to the department of Chocó. Description of 47 municipalities that belong to 10 rural areas defined as GMD and their departments, which represent a region with mostly malaria endemic areas. Source: UPME (2005).

*Data sources* - Data was obtained from sources such as the official national surveillance system [National Health Institute (SIVIGILA)], reports from the Ministry of Mines and Energy, the Colombian Mining Information System, and the Office of the Ombudsman (Defensoría del Pueblo de Colombia 2010).

**Fig. 1A:**
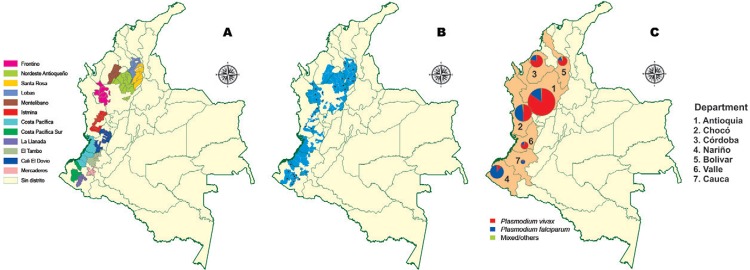
: gold-mining distribution in malaria endemic areas in Colombia. Name and geographic gold-mining districts (GMD) distribution in Colombia. Source: modified from simco.gov.co/Simco/Portals/0/mapaDistritosMineroscolombia2008.pdf; B: gold-mining production units or municipalities (spot) by GMD. Source: modified from Cuales son los distritos mineros de Colombia? (simco.gov.co/simco/Politicasdelsector/MejoramientodelaProductividadyCompetitividad/Gesti%C3%B3ndelosDistritosMineros/tabid/86/Default.aspx); C: total morbidity of malaria distribution in Colombia by parasite species in 2010-2013.

*Mining districts* - “Mining districts” denote areas where mining is performed according to the rules and regulations established by local miners. There are no limits to their territory and their boundaries can change. Ten rural mining districts were selected from departments with the highest gold production. All municipalities were located on alluvial areas or had other environmental factors that promoted ecological niches for the development of malaria vectors (Fig. 1A).

The study population included 1,250 Afro-Colombian communities, with an estimated population of ~270,000 people and various indigenous groups that formed approximately 4-5% of the total population. These regions have extreme poverty according to national standards, and have the worst national social and economic indicators.

*Data analyses* - An Excel database was designed and validated to store the collected information. Epidemiological and mining variables defined for the study were: number of total cases of malaria from 47 municipalities in 10 gold-mining districts (GMD), parasite species, population at risk, annual gold production in towns, and mining districts, over a study period from 2010-2013. The API was calculated using the total number of malaria cases in each municipality and the population at risk for each year in the study period and was reported per 1,000 inhabitants. Statistical tests were performed to look for correlation between annual gold productions in Colombia (estimated in tons) APIs in different mining districts. Potential malaria vectors described here are based on published studies (Montoya-Lerma et al. 2011). Image files of maps in Portable Network Graphics format were used as a data source to create new maps. These were superimposed using GNU Image Manipulation Program v.2.8.14 (an open-source raster graphics editor used for image retouching and editing).

Databases were refined according to the recommendations of the monitoring system (SIVIGILA) that included address, validation rules, variable code, date of service, type identification, identification number, primary data generating units and information units, which permitted assessment of duplicate cases. A plan of analysis of the variables was established to calculate absolute and relative frequencies and perform univariate analysis, bivariate correlation, or R square (R^2^) between gold production (ton/year/GMD) and API with statistical significance tests. Statistical analyses were performed using a database on Excel 2013 and PRISMA GraphPad Prism v.6.01.

## RESULTS

*Mining districts in Colombia* - According to the classification by the Unit of Planning of Mining and Energy in Colombia, the departments with highest gold production are Antioquia, Córdoba, Bolívar, Chocó, Nariño, Cauca, and Valle, which contributed to 89.3% (270,753 cases) of the national malaria incidence from 2010-2013, of which 31.6% came from mining areas (Fig. 1A-C). The 2011 census of mining activities in Colombia reported 4,134 gold mines and only 550 had official owner-titles. Of the 4,134 mines, 2,976 (72%) were located on the flank of the western mountains in the departments of Antioquia, Bolívar, and Chocó (Fig. 1B). The gold mines below this range in the Pacific lowlands of Valle del Cauca and Nariño are mostly illegal.

*Malaria in gold-mining areas of Colombia* - During the study period, there was a decreasing trend in malaria in Colombia from ~117,000 cases in 2010, with an API of 11.5/1,000 inhabitants, to ~60,000 in 2013, with an API of 4.95/1,000 inhabitants. In this period there was a reduction in malaria incidence of ~51.3% (Table II). This trend was also observed in the mining regions with a few exceptions, including the districts of San Martín de Loba (Bolívar) and South Pacific Coast (Nariño), where malaria prevalence has been stable or has increased over the same period (Table II). However, at department level, in 2012-2013 the departments of Antioquia and Chocó had the highest API of greater than 20/1,000 inhabitants, which were followed by Nariño, with an API of 10.1/1,000 inhabitants. In 2013, 88.8% of national cases were reported from Antioquia (39.5%), Chocó (24.8%), Córdoba (5.4%), Nariño (10.1%), Bolívar (5.8%), Valle del Cauca (1.7%), and Cauca (1.6%). Most infections (64.9%) were caused by *P. vivax*, 33.6% by *P. falciparum*, and 1.4% were mixed infections.

**TABLE II:** Distribution of *Plasmodium* spp causing malaria in Colombian gold-mining district between 2010-2013

Mining district (department)	2010		2011		2012		2013	
			
	*P. falciparum* n (%)	*P. vivax* n (%)	*P. falciparum* n (%)	*P. vivax* n (%)	*P. falciparum* n (%)	*P. vivax* n (%)	*P. falciparum* n (%)	*P. vivax* n (%)
Santa Rosa (Bolívar)	91 (0.09)	256 (0.25)	36 (0.10)	258 (0.40)	9 (0.02)	220 (0.40)	12 (0.02)	261 (0.49)
Itsmina (Chocó)	325 (0.31)	640 (0.61)	124 (0.21)	200 (0.89)	102 (0.19)	217 (0.39)	63 (0.12)	91 (0.17)
Costa Pacífica (Cauca and Valle del Cauca)	1,114 (1.07)	3,388 (3.24)	861 (1.49)	1,457 (2.52)	233 (0.42)	854 (1.56)	525 (0.98)	618 (1.15)
Costa Pacífica Sur (Nariño)	70 (0.07)	28 (0.03)	78 (0.13)	13 (0.02)	827 (1.51)	71 (0.13)	734 (1.36)	93 (0.17)
San Martín de Loba (Bolívar)	12 (0.01)	144 (0.14)	14 (0.02)	160 (0.28)	37 (0.07)	364 (0.66)	20 (0.04)	516 (0.96)
La Llanada (Nariño)	36 (0.03)	4 (0.01)	5 (0.01)	5 (0.01)	17 (0.03)	1 (0.01)	3 (0.01)	6 (0.01)
Mercaderes (Cauca and Nariño)	4 (0.01)	8 (0.01)	-	1 (0.01)	2 (0.01)	2 (0.01)	-	1 (0.01)
Frontino (Antioquia)	89 (0.09)	892 (0.85)	8 (0.01)	414 (0.72)	19 (0.03)	576 (1.05)	23 (0.04)	823 (1.53)
Northeast Antioqueño (Antioquia)	5,952 (5.70)	15,099 (14.45)	1,793 (3.10)	13,415 (23.22)	1,053 (1.92)	8,506 (15.54)	1,297 (2.41)	6,238 (11.60)
Montelíbano (Córdoba)	2,292 (2.19)	4,147 (3.97)	655 (1.13)	2,900 (5.02)	222 (0.41)	1,644 (3.004)	294 (0.55)	1,185 (2.20)

Total	9,985 (9.60)	24,606 (23.60)	3,574 (6.20)	18,823 (32.60)	2,521 (4.60)	12,455 (22.76)	2,971 (5.52)	9,832 (18.28)

Source: National Health Institute of Colombia using a sispro data base platform (sispro.gov.co).

Before 2010, the mining districts with the highest APIs were northeast Antioquia, Montelíbano (Córdoba), and Santa Rosa (Bolívar), however since 2013, Antioquia, San Martín de Loba (Bolívar), and the South Pacific Coast have had the highest. Although the API in Colombia has decreased over the last five years, it has increased by more than 50% in the mining districts of San Martin de Loba, Costa Pacífica South, and northeast regions of Antioquia (Table III).

**TABLE III:** Gold production, malaria cases, and annual parasite index (API) on gold-mining district

Mining district (department)	2010			2011			2012			2013		
			
	Cases (n)	Gold (ton)	API (1,000/h)	Cases (n)	Gold (ton)	API (1,000/h)	Cases (n)	Gold (ton)	API (1,000/h)	Cases (n)	Gold (ton)	API (1,000/h)
Santa Rosa (Bolívar)	422	6.4	9.64	336	6.9	9.64	248	7.4	7.18	277	7.9	8.09
Itsmina (Chocó)	986	1.6	4.81	333	1.6	4.81	328	1.6	4.70	156	1.6	2.22
Costa Pacífica (Cauca and Valle del Cauca)	4,539	-	5.29	2,330	-	5.29	1,096	-	2.45	1,157	-	2.54
Costa Pacífica Sur (Nariño)	98	0.4	1.31	91	0.4	1.31	901	0.4	12.73	827	0.4	11.46
Distrito San Martín de Loba (Bolívar)	189	1.4	4.47	179	1.6	4.47	426	1.8	10.52	541	2	13.21
Distrito La Llanada (Nariño)	41	0.8	0.05	10	0.9	0.12	18	1.0	0.21	9	1.1	0.10
Distrito Mercaderes (Cauca and Nariño)	12	0.1	0.01	1	0.1	0.01	4	0.1	0.05	1	0.2	0.01
Frontino (Antioquia)	981	0.4	4.86	424	0.4	4.86	599	0.4	6.83	850	0.5	9.65
Nordeste Antioqueño (Antioquia)	21,242	23.2	182.63	15,305	29.1	182.63	9,612	29.9	113.43	7,589	28.6	88.59
Montelíbano (Córdoba)	6,493	3.9	20.18	3,583	3.9	20.18	1,875	3.9	9.78	1,494	3.3	8.08

Total	35,003	38.2	19.37	22,592	44.9	19.37	15,107	46.5	12.67	12,901	45.6	10.77

Source: INCOPLAN SA (2011).

The highest APIs in the country were found in the municipalities in Antioquia [El Bagre (188.2/1,000), Segovia (137.0/1,000), Zaragoza (99.9/1,000), Remedios (28.9/1,000), Mutatá (25.1/1,000), and Santa Barbara (20.1/1,000)] followed by municipalities in Bolívar [San Jacinto de Achí (34.4/1,000) and Tiquisio (32.0/1,000)], Córdoba [Puerto Libertador (26.2/1,000)], and Bolívar [Santa Rosa del Sur (13.1/1,000)]. In contrast, the municipality from the districts of Istmina and Costa Pacífica showed a reduction per 1,000 habitants at risk (Table III).

Despite the recent malaria reduction in most mining districts, they still contribute considerably to the national prevalence (Table II). Correlation analyses indicated that there are two groups of mining districts: those with high gold production methods with large numbers of cases and high APIs, and those with low gold production with a low number of malaria cases reported (Table III).

**Fig. 2:**
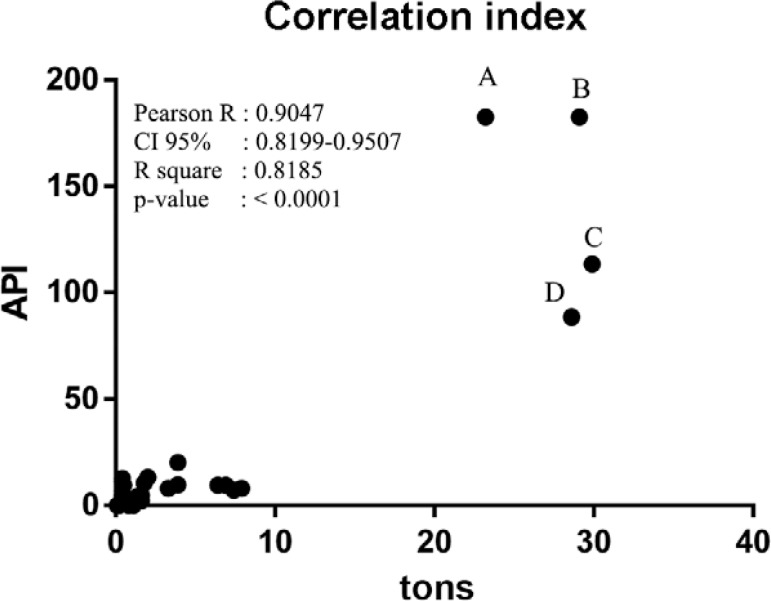
: correlation between annual parasite index (API) and gold-mining district (GMD) production. The increased value of API is explained by tons of gold produced in a GMD (northeast Antioquia) from 2010-2013 (A: 2010; B: 2011; C: 2012; D: 2013). Populations with greater than 50 tons of gold produced had higher risk of malaria infection than those with a lower production. CI: confidence interval.

Although the linear model is not the most appropriate, a R^2^ determination coefficient of 0.69 indicated that 69% of the variance in number of cases was explained by gold-mining production for the analysed time interval. Adjusting the correlation described by the size of the population at risk terms or API (positive thick smear/1,000 inhabitants) resulted in a correlation of 0.90 where R^2^was equal to 0.81. This also indicated that the variance of malaria cases in mining areas was explained by the magnitude of the activity and mining per ton (Fig. 2).

Ecological studies found that *Anopheles darlingi*, *Anopheles albimanus*, and *Anopheles nuñeztovari* are the primary malaria vectors in mining areas (Montoya-Lerma et al. 2011). In the department Córdoba (municipalities of Montelíbano and Puerto Libertador) *An. darlingi* and *An. nuñeztovari* were found infected with *P. vivax,* (Gutiérrez et al. 2009). Whereas in Chocó*An. darlingi* was the major vector of *P. falciparum*, however, *An. albimanus* and *An. darlingi* were also found infected with *P. falciparum*and with *P. vivax* in Chocó and Valle del Cauca (Buenaventura), respectively. Other species are considered to be secondary vectors, such as*Ano- pheles pseudopunctipennis* and *Anopheles neivai*, which are important vectors of human malaria transmission in the Pacific coastal areas of Colombia (Sinka et al. 2010).

## DISCUSSION

In mining districts, the greatest contribution to the national malaria incidence came from the northeast Antioquia district (Antioquia), Pacific Coast district (Chocó), South Pacific Coast and La Llanada districts (Nariño), Montelíbano district (Córdoba), and San Martín de Loba district (Bolívar). Although one third (36%) of the cases recorded nationally were reported from mining areas, these figures may be underestimated due to population migration and under-recording of malaria cases in areas with illegal mining activity. Nevertheless, in spite of the 50% reduction in malaria cases between 2010-2013 at national level, this study indicates that mining plays an important role in the maintenance of malaria transmission and imposes an important barrier to malaria elimination, particularly in these regions.

In Colombia, two types of gold mine exploitation related to malaria exist in both legal and illegal mining. One of them is the alluvial type with low rates of malaria cases. In the Colombian Pacific Coast, particularly, miners practice the artisanal mining called *barequeo* (gold-panning), consisting of traditional manual gold extraction using craft devices (MinAmbiente 2001, Suárez 2011).*Barequeo* has traditionally been a single-person operation for extracting minerals in small quantities. The other type of mining is a more modern type of gold-mining extraction, for example in Antioquia *vetas* and “open sky”, and was associated with a high number of malaria cases in the study period. This method which uses bulldozers and dredges that have helped mechanise this activity. In addition the clandestine nature of gold-mining, its poor control by authorities has led to poor planning and structure with little legalisation of this activity throughout of the country (Suárez 2011, Güiza & Aristizabal 2013, Semana 2015). Thus, it was associated with many cases of malaria during the study period.

**Fig. 3:**
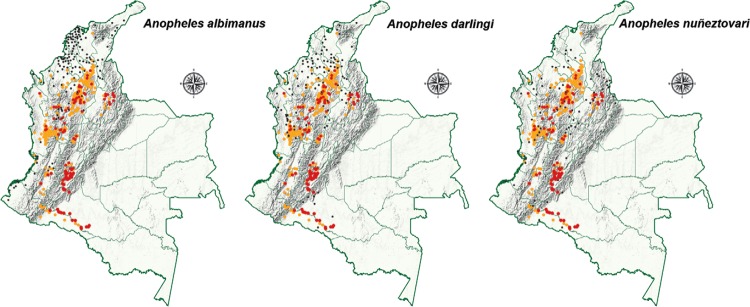
: distribution of *Anopheles* species in gold-mining areas of Colombia. Illegal gold mines are shown in yellow and legal mines are shown in red. Dark dots show the distribution for the indicated mosquito species in each map. Source: modify from Montoya-Lerma et al. (2011).

Although *P. vivax* represents ~68% of malaria cases recorded nationally, *P. falciparum* presented a high prevalence (46.7%) in the Pacific Coast mining districts (Fig. 1C). In these districts, most of the population are of African descent and therefore the Duffy negative (Fy-) blood group is highly prevalent. The absence of the Fy- blood group affects the rate of *P. vivax* infections, as it offers protection again *P. vivax* blood infection (Herrera 2005).*An. darlingi* and *An. albimanus* are commonly in these districts and breed in within “open sky” mining. These two vectors maintain transmission which occurs predominantly in the first part of the night. *P. vivax* infections, which comprise 24-40% of the total of cases of malaria in mining areas are transmitted by *An. darlingi*, *An albimanus*, *Anopheles calderoni*, and *An. nuñeztovari* and this has been observed in the Pacific Coast and South Pacific mining districts and the departments of Nariño, Cauca, and Valle del Cauca (Fig. 3) (Gutiérrez et al. 2008, 2009,Montoya-Lerma et al. 2011).

Measures such as reforestation with landscape recovery, vector control activities, better screening for malaria, increased use of repellents, easier access to quality care, and treatment with gametocytocides need to be evaluated for inclusion in malaria elimination programs in mining areas.

ACD offers entire population screening in an area at a given time; however, this method detects only symptomatic cases (Schellenberg et al. 2003, Snow et al. 2005). Due to the high mobilisation of the population in mining areas, the case monitoring process would be interrupted and cases originating in these areas could be labelled as imported cases elsewhere. Thus, ACD is not viable in this context.

Mining populations consist somewhat of malaria susceptible migrants from nonmalaria-endemic areas, who are at great risk of malaria due to the lack of immunity. When this same population returns to their place of origin, they take their parasite infections with them, thus introducing new infections in malaria-naïve communities (Khasnis & Nettleman 2005, Barbieri & Sawyer 2007). These populations migrate frequently, and if infected, have the potential to rapidly disseminate different *Plasmodium* strains to neighbouring regions (Khasnis & Nettleman 2005).

Reactive, proactive, and aggressive case detection should be the approach for the detection of asymptomatic cases; however, this methodology would only be viable to reduce transmission rates if more sensitive and specific methods such as polymerase chain reaction-based are used to detect cases with low parasite densities (Macauley 2005, Feachem et al. 2010). The frequency of asymptomatic infections from active searches has not been established to be included in the Colombian National Malaria Control Program. These cases can be very significant in regions with a high frequency of infections reported in the working population whose role in malaria transmission remains unknown as it was evidenced in Buenaventura mining district. Moreover, severe malaria is highly endemic in rural communities that are within close proximity to gold-mining extraction activities as seen in the departments of Chocó and Nariño. In these areas, ongoing studies indicate that the prevalence of complicated malaria cases is 0.5%.

Colombia’s annual gold production is expected to increase significantly with the discovery of new mining areas in the Serrania de San Lucas (Bolívar). The resulting association between gold-mining and malaria found in this study underscores an urgent need for improving malaria prevention and control measures in gold-mining areas by government entities and nongovernmental organisations.

This malaria study was conducted to establish the epidemiological parameters based on officials reports from health and mining ministry, in order to review activities to control malaria and other potential vector borne diseases in the area. However, it requires an upgrade of the current and actual statistics with a more in-depth study of the socio-cultural, demographic, and ecological characteristics of mining areas in relation to malaria.

In spite of a reduction of 50% in malaria cases between 2010-2013 in Colombia, a significant proportion of the cases (36%) are related to gold-mining activities. The legal and illegal mining areas are located in regions with a high prevalence of malaria, where malaria vectors are also present. Therefore, mining plays an important role in the maintenance of malaria transmission and is an important barrier to malaria elimination in this region. Even though malaria is decreasing in some nonmining endemic departments, it is increasing in the mining districts, such as Costa Pacífica Sur, in Nariño, and San Martín de Loba, in Bolívar. Aggressive case detection followed by prompt treatment is urgently required to diminish the negative influence of mining regions on malaria transmission. In departments with the highest gold-mining production units, Antioquia, Bolívar, Córdoba, and Chocó, elimination strategies should focus specifically on gold-mining areas.
